# Computation and molecular pharmacology to trace the anti-rheumatoid activity of *Angelicae Pubescentis Radix*

**DOI:** 10.1186/s12906-022-03769-w

**Published:** 2022-11-26

**Authors:** Junhao Zhang, Rui Wang, Xiao Liang, Hao Tian Bai, Ya Lan Li, Shuhui Sun, Qianqian Zhang, Jing Yang

**Affiliations:** 1 College of Pharmacy, Heilongjiang University of Traditional Chinese Medicine, Harbin, 150000 China; 2 Basic Medical College, Heilongjiang University of Traditional Chinese Medicine, Harbin, 150000 China

**Keywords:** *Angelicae Pubescentis Radix*, Cell experiment, Molecular docking technology, Network pharmacology, Rheumatoid joint, Signaling pathway

## Abstract

**Background:**

The mechanism of action of *Angelicae Pubescentis Radix* in rheumatoid arthritis treatment is complex; the pathways and protein targets involved remain unclear. This study predicted the targets and signaling pathways of *Angelicae Pubescentis Radix* for rheumatoid arthritis treatment using network pharmacology and molecular docking technology and clarified its mechanism of action using in vitro cellular experiments.

**Methods:**

*Angelicae Pubescentis Radix* active components and related targets were retrieved from the traditional Chinese medicine systems pharmacology database. All human proteins were mined from the global protein database, and the network of active components and targets of *Angelicae Pubescentis Radix* was drawn using Cytoscape 3.7.1. GeneCard, Online Mendelian Inheritance in Man, and DrugBank databases were used to mine rheumatoid arthritis-related genes. Metascape was used for Gene Ontology function analysis and Kyoto Encyclopedia of Genes and Genomes enrichment pathways. β-sitosterol’s molecular docking was determined using AutoDock Tools; pathway verification was performed in the Kyoto Encyclopedia of Genes and Genomes database, and the verified genes were input into the Human Protein Atlas database to observe the expression levels in various human body tissues.

**Results:**

Eight main active components were screened out of *Angelicae Pubescentis Radix* from the traditional Chinese medicine systems pharmacology database, and 60 targets related to major active ingredients were obtained. Forty-two core pathogenic rheumatoid arthritis-related genes were screened from GeneCard and other related databases. The enrichment of the Kyoto Encyclopedia of Genes and Genomes pathway included the vascular endothelial growth factor signaling pathway that proved to be the decisive pathway for rheumatoid arthritis treatment by a high degree value. In vitro experiments confirmed that *Angelicae Pubescentis Radix* mainly regulated cell proliferation and survival through the vascular endothelial growth factor signaling pathway and showed significant therapeutic effects on rheumatoid arthritis. The prostaglandin endoperoxide synthase 2 gene was associated with rheumatoid arthritis via pathway verification and monitoring of human gene expression levels.

**Conclusions:**

The mechanism of the multi-component, multi-target, and multi-channel treatment of rheumatoid arthritis via *Angelicae Pubescentis Radix* was explored using network pharmacology and molecular docking technology, providing new thinking and research directions for future rheumatoid arthritis treatment using *Angelicae Pubescentis Radix.*

**Supplementary Information:**

The online version contains supplementary material available at 10.1186/s12906-022-03769-w.

## Background

Rheumatoid arthritis (RA) is a chronic autoimmune disease with characteristics of symmetry, progression, and invasion [1]. It has always been a serious disease threatening human health. The global incidence of RA is approximately 1%, and its incidence in China is 0.3–0.4% [2]. The current treatment methods for RA include medications and surgical treatment. Medications have decreased efficacy and severe adverse reactions after long-term use, including cardiotoxicity, immunosuppression, severe infection, and other adverse reactions [2], while surgical treatment results in the expansion of wounds, requires postoperative recovery, and increases the financial burden on families. However, traditional Chinese medicines can be used to treat RA via multi-level, multi-component, and multi-target methods, and they can counteract the toxicity and side effects of conventional medicine. Several studies have reported that Chinese medicine promotes cartilage repair; increases bone mineral density; inhibits vascular proliferation; enhances fibrinolysis, anti-inflammation, and analgesia; and regulates immunity [3–5]. Therefore, the unique advantages of traditional Chinese medicine for the treatment of RA, such as decreased toxicity, fewer adverse effects, and good curative effects, have inspired new treatment methods. While the development, metastasis, and invasion of RA are complex processes, traditional Chinese medicine can play an important role in the treatment of RA.

*Angelicae Pubescentis Radix*, the dried roots of *Angelica biserrata* C.Q.Yuan & R.H.Shan (a synonym for *Angelica pubescens f. biserrata* R.H. Shan & C.Q. Yuan in Chinese Pharmacopoeia), is currently used as a medicine. It has recently been verified to have anti-inflammatory, analgesic, sedative, and anti-tumor effects, as well as the short-term effects of lowering blood pressure and inhibiting platelet aggregation [6]. *Angelicae Pubescentis Radix* is currently used to treat soreness of the waist and knees, rheumatism, and arthralgia. It is commonly used for the treatment of RA. However, its therapeutic mechanism is unclear, and further research regarding its mechanism is needed. Therefore, an exploration of the active components of *Angelicae Pubescentis Radix* is necessary, and its mechanism for the treatment of diseases such as RA should be determined to provide a basis for future pharmacological research.

The mechanism of action of *Angelicae Pubescentis Radix* for the treatment of RA can be explored at the molecular level through network pharmacology and molecular docking technology. The network of the active components, targets, and dredging collaterals of *Angelicae Pubescentis Radix* can be constructed and clarified via multiple aspects, revealing its pharmacodynamic components and mechanism of action for the treatment of RA [4]. This would provide an accurate and objective basis for future research and clinical applications.

**Fig. 1:**
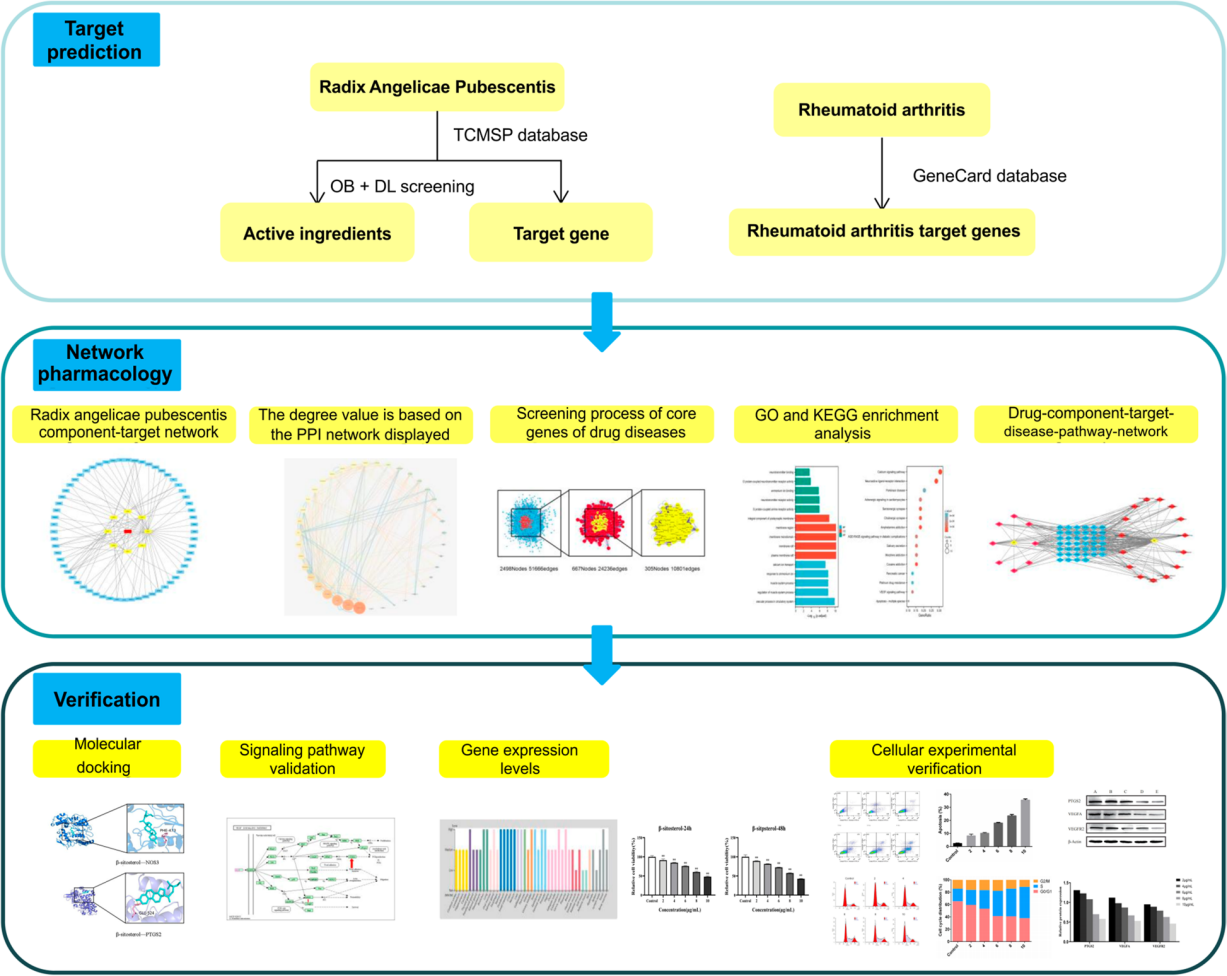
Workflow chart. TCMSP, traditional Chinese medicine systems pharmacology; OB, Oral bioavailability; DL, Druglikeness; PPI, Protein-protein interaction; GO, Gene Ontology; KEGG, Kyoto Encyclopedia of Genes and Genomes

This study aimed to predict the targets and signaling pathways of *Angelicae Pubescentis Radix* for the treatment of RA using network pharmacology and molecular docking technology. In addition, we aimed to clarify and verify the mechanism of action using in vitro cellular experiments. Fig. 1 shows the overall process of the study.

## Methods

### Identification of candidate components in Angelicae Pubescentis Radix

The medicinal components of *Angelicae Pubescentis Radix* were retrieved from the traditional Chinese medicine systems pharmacology database and analysis platform (TCMSP) [5].

### Screening strategy for bioactive components of Angelicae Pubescentis Radix

Traditional Chinese medicines (TCMs) need to be distributed rapidly in the process of absorption, distribution, metabolism, and excretion (ADME). In ADME, oral bioavailability (OB) is one of the most representative pharmacokinetic parameters; thus, substances with OB ≥30% have higher druggability.

Druglikeness (DL) is used as a qualitative concept to estimate the drug properties of molecules in drug design. The DL index can be used to quickly screen active substances, and substances with DL ≥0.18 are considered to have druggability.

Therefore, the compounds of *Angelicae Pubescentis Radix* with OB ≥30% and DL ≥0.18 were chosen as the active ingredients in this experiment.

### Prediction of active component targets of Angelicae Pubescentis Radix

All human protein and gene data were downloaded from the global protein resource database (Uniprot, http://www.Uniprot.org). Active component targets retrieved from the TCMSP database were compared with the Uniprot database to obtain standard gene names.

### Collection and arrangement of RA targets

The human genes (GeneCard, http//www.GeneCard.org), Online Mendelian Inheritance in Man (OMIM, http://www.omim.org), and DrugBank (http://www.go.drugbank.com) databases were searched using the keyword “rheumatoid arthritis” [7] to obtain target genes of RA. The results were collated for screening and comparison to select the intersection. The data collated from the Uniprot database were matched with the intersection to obtain the targets for RA.

### Construction of the protein-protein interaction network

The target genes of the components of *Angelicae Pubescentis Radix* and RA were intersected, and the intersected genes were input into the STRING online database (https://string-db.org) to construct a target network using the “organization” option. The minimum required interaction score was set to have medium reliability of 0.400. No maximum number of associates was set. The association importance and interaction of each node were determined using the difference in the degree values.

### Gene ontology and genomes pathway enrichment analysis for RA-related targets of Angelicae Pubescentis Radix

The Metascape database and were used to conduct a functional enrichment analysis of the Gene Ontology (GO) and a pathway enrichment analysis of the Kyoto Encyclopedia of Genes and Genomes (KEGG) pathway with the background set as *Homo sapiens* to clarify the roles of the target proteins in the gene functions and signaling pathways [8–11]. Functional annotation and pathways of potential genes were visualized using biological processes (BP), molecular functions (MF), and cellular components (CC). An adjusted *P*-value of < 0.05 was set as the threshold value, and statistical significance was set at a *P*-value < 0.05.

### Construction and analysis of network

To further visualize the molecular mechanisms of the active ingredients against RA, the obtained data were organized into network and type files that were imported into Cytoscape version 3.7.1 (NIH Biomedical Technology Research Center, Bethesda, MD, USA), an open-source software available at http://cytoscape.org [12]. The software generated a network of active ingredients of the drugs, as well as target and disease pathways. In a graphical network, nodes represented components, targets, and pathways, while interactions between nodes were represented by edges. The “analyze network” function was used to calculate the degree between the active component and target. Larger degree values represented more important active components.

### Molecular docking analysis

Molecular docking includes three steps: (a) preparation of ligands, (b) preparation of macromolecules (targets) and determination of their active sites, and (c) ligand-target docking.

Here, a two-dimensional structure of β-sitosterol was obtained using PubChem software (National Institutes of Health, Bethesda, MD, USA) and stored in the SDF format that was processed using ChemBio3D (CambridgeSoft, Waltham, MA, USA) to obtain a three-dimensional structure with minimum energy.

The target’s crystal structure was obtained from the Protein Data Bank, and the water molecules and hydrogenation were removed using PyMOL software (Schrodinger Company, New York, NY, USA).

Autodock Tools software (Scripps Research Institute, La Jolla, CA, USA) was used to convert the three-dimensional structure of β-sitosterol and crystal structure of the target into the pdbqt format. Molecular docking was conducted with β-sitosterol, a key active component in *Angelicae Pubescentis Radix*, and the 10 genes with the highest degree values in the protein-protein interaction (PPI) network. Molecular docking was assessed using Autodock Vina software (Scripps Research Institute) to evaluate the binding of β-sitosterol and the target based on the binding energy standards, and visual processing was performed using PyMOL software.

### Experimental verification

#### Cells

MH7A rheumatoid arthritis fibroblast cells (lot number 21112414) were purchased from Beina Bio (Hunan, China).

#### Drugs and reagents

Dulbecco’s modified eagle medium (Art. No.10–013-CVRC, Corning, New York, NY, USA); β-sitosterol (Batch No. Y22A10C85758, Shanghai Yuanye Biotechnology Co., Ltd., Shanghai, China), fetal bovine serum (Art. No.04–007-1a, Biological Industries, Kibbutz Beit Haemek, Israel), phosphate buffer saline (PBS; Art.No. WH0112201 911 XP, Procell, Wuhan, China), pancreatin (Art. No.143188, Biosharp, Hefei, China), DMSO (Tianjin Fuyu Fine Chemical Co., Ltd., Wuching District, China), MTT (Art. No. M8180, Beijing Suleibao Technology Co., Ltd., Beijing, China), a BCA kit (Lot No.20210922, Bio-Swamp Life Science Lab, Wuhan, China), an ECL high-sensitivity chemiluminescent solution kit (Batch No.: GC 10AA0033, Biological Engineering Co., Ltd., Shanghai, China), β-actin (Batch No.: F200040, Abways Technology, Shanghai, China), VEGFA (Batch No. 83 m8093, Affinity Biosciences, Beijing, China), PTGS2 (Batch No. 86F4760, Affinity Biosciences), VEGFR2 (Batch No. 84 g5912, Affinity Biosciences), and horseradish peroxidase-labeled goat anti-rabbit IgG secondary antibody (Batch No. F300405, Abways Technology) were used in this study.

#### Instruments

A CO_2_ incubator (Wiggins WCI-180; Beijing Sanyi Experimental Instrument Institute, Beijing, China), clean bench (SW-CJ-2FD; SDT Scientific Instrument Co., Ltd., Shanghai, China), centrifuge (TD5; Shanghai Lu Xiangyi Centrifuge Instrument Co., Ltd., Shanghai, China), microplate reader (SPARK 10 M; TECAN, Männedorf, Switzerland), electrophoresis instrument (DYCZ-2DN; Beijing Liuyi Biotechnology Co., Ltd., Beijing, China), decoloring shaker (WD-9405F; Beijing Liuyi Biotechnology Co., Ltd), and chemiluminescence analyzer (WD-9423B; Beijing Liuyi Biotechnology Co., Ltd) were used in this study.

#### MTT cell proliferation assay

The proliferation of MH7A cells was detected using the MTT cell proliferation assay. Cells in the logarithmic phase were inoculated on culture plates and incubated for 24 h and 48 h at 37 °C with 5% CO_2_. The cells were divided into blank, control, and medication groups. When the cell density reached 80%, β-sitosterol was added to the medication group at gradient concentrations (2 μg/mL, 4 μg/mL, 6 μg/mL, 8 μg/mL, and 10 μg/mL). After 24 h, 5 μg/mL MTT solution was added, and the cells were cultured for 4 h, after which the supernatant in the wells was discarded, and 150 μL DMSO was added to each well. The solution was shaken on a shaking table in the dark for 10 min until the crystals were completely dissolved. The optical densities (ODs) of the well plates were measured using a microplate reader at 570 nm and compared with the OD of the control group to determine the relative cell activity.

#### Detection of cell cycle and apoptosis rate via flow cytometry

The effects of β-sitosterol on the cell cycle and apoptosis were detected using flow cytometry. MH7A cells in the logarithmic phase were inoculated into 96-well plates and were incubated with gradient concentrations of β-sitosterol for 24 h. Then, the cells were digested with trypsin, centrifuged at 1500 r/min for 5 min, and washed with PBS. The supernatant was discarded after centrifugation, and the cells were fixed with 2-mL 75% ethanol at 4 °C for 12 h. The cells were centrifuged, the supernatant was removed before two washes with PBS were conducted, and 500 μL of propidium iodide and ribonuclease mixture was added. For the apoptosis analysis, the cell concentration was adjusted to 1 × 10^5^/L, and 100 μL of binding buffer, 5 μL of annexin V-phycoerythrin, and 5 μL of 7-amino-actin D were added.

#### Western blot assay to determine protein content

Cells were lysed with radioimmunoprecipitation assay lysis buffer on ice for 30 min and centrifuged at 1200 r/min at 4 °C. The supernatant was used for protein quantitative detection via the BCA method. The protein samples were denatured and stored at − 20 °C. Sodium dodecyl sulfate-polyacrylamide gel electrophoresis was conducted, followed by membrane transfer via blocking with TBST containing 5% defatted milk powder for 2 h. The membrane was incubated with antibodies against VEGFA, VEGFR2, or PTGS2 for 12 h at 4 °C and then washed with TBST (three washes, 15 min each). The membranes were then incubated with secondary antibodies (horseradish peroxidase-labeled) for 2 h at room temperature and were washed again with TBST (three washes, 15 min each). The membrane was developed using the ECL chemiluminescent color method and ImageJ software (National Institutes of Health, Maryland, USA).

### Statistical analyses

Experimental data are shown as means±standard deviations. SPSS 22.0 (IBM Corp., Armonk, NY, USA) software was used for statistical analysis. One-way analysis of variance was used for multi-group comparison, and the t-test was used for inter-group comparison. *P* < 0.05 was considered to indicate a statistically significant difference.

## Results

### Selection of effective components of Angelicae Pubescentis Radix

We selected components with potential biological effects according to the following criteria: ADME characteristics of OB ≥30% and DL ≥0.18. After selection, the maximum and redundant data were removed, and 99 chemical components of *Angelicae Pubescentis Radix* were identified. Eight active components met the screening criteria, which mainly comprised coumarin compounds (Ammidin, Isoimperatorin, O-Acetylcolumbianetin, Angelol D), sterol compound (β-sitosterol), polysaccharide compounds (Angelicone nodakenin), and so on (Table 1).

**Table 1:** Active ingredients of *Angelicae Pubescentis Radix*

Molecular ID	Component name	OB (%)	DL (%)	Abbreviation
MOL001941	Ammidin	34.55	0.22	DY1
MOL001942	Isoimperatorin	45.46	0.23	DY2
MOL008583	β-sitosterol	36.91	0.75	DY3
MOL003608	O-Acetylcolumbianetin	60.04	0.26	DY4
MOL004777	Angelol D	34.85	0.34	DY5
MOL004778	[(1R,2R)-2,3-dihydroxy-1-(7-methoxy-2-oxochromen-6-yl)-3-methylbutyl] (Z)-	46.03	0.34	DY6
MOL004780	2-methylbut-2-enoate	30.99	0.19	DY7
MOL004792	Angelicone nodakenin	57.12	0.69	DY8

Abbreviations: *OB* Oral bioavailability, *DL* Druglikeness

### Components of Angelicae Pubescentis Radix and target network construction

**Fig. 2:**
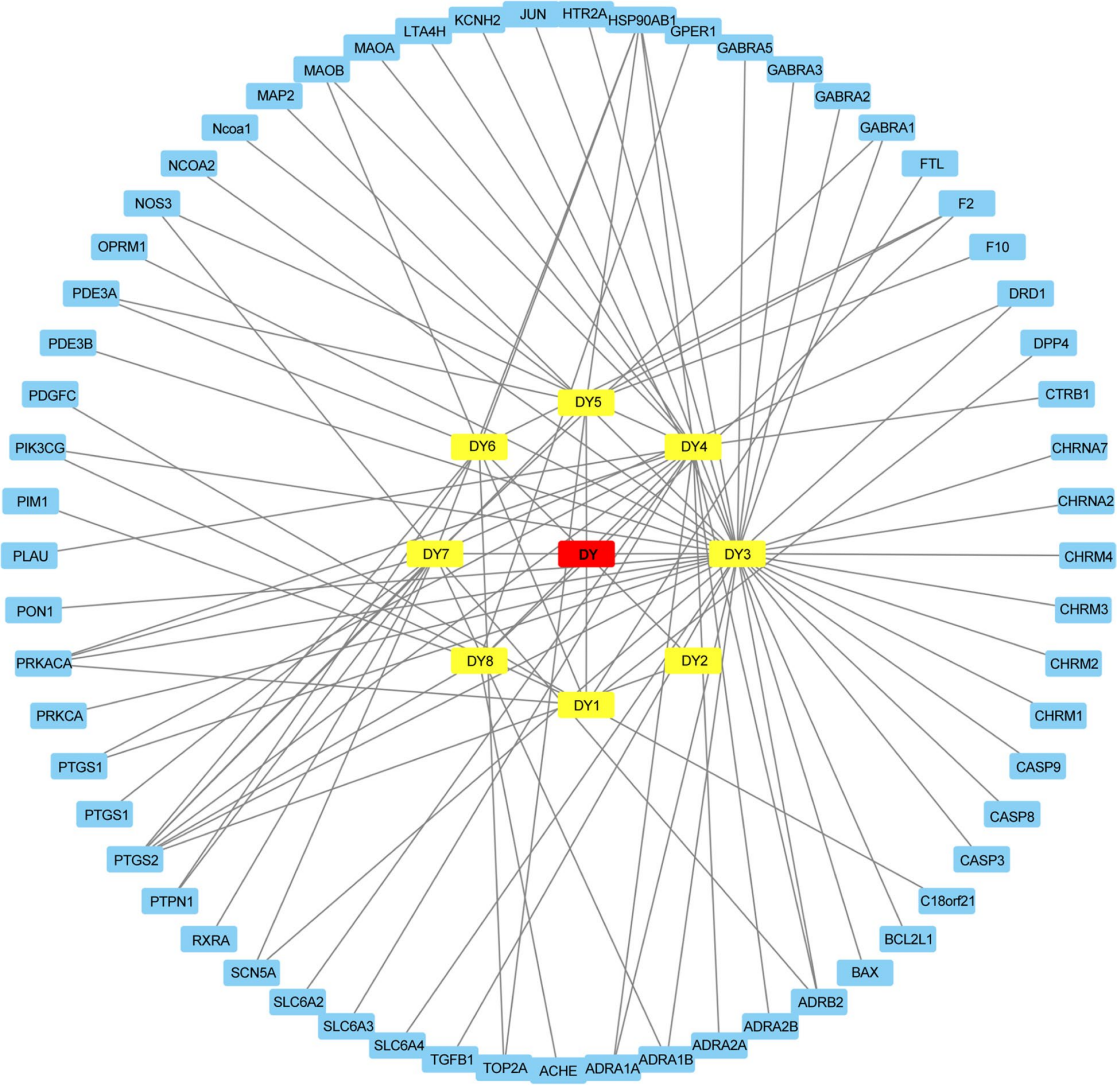
Essential components and target network of *Angelicae Pubescentis Radix*

Sixty target proteins were screened out based on one-to-one correspondence with the eight active components of *Angelicae Pubescentis Radix* (Fig. 2).

In the network interaction diagram, red, yellow, and blue rectangles represent *Angelicae Pubescentis Radix*, the active ingredient, and the target, respectively.

### Identification of RA genes

Among the eight candidate bioactive components, 4735 RA-related targets were identified in the GeneCard database. After eliminating the overlaps, the obtained targets were further analyzed by Uniprot database and 550 targets were identified in the DrugBank database. The genes that interacted with the active components of *Angelicae Pubescentis Radix* were intersected to identify the common genes. Therefore, 60 target proteins from 8 active components in *Angelicae Pubescentis Radix* were related to RA, with detailed information of these 60 targets reported in Table 2.

**Table 2:** Potential central genes of Angelicae Pubescentis Radix against rheumatoid arthritis (RA)

Gene	Full name
CHRM1	Muscarinic acetylcholine receptor M1
PTGS2	Prostaglandin G/H synthase 2
GABRA1	Gamma-aminobutyric acid receptor subunit alpha-1
DPP4	Dipeptidyl peptidase IV
PIK3CG	Phosphatidylinositol-4,5-bisphosphate 3-kinase catalytic subunit, gamma isoform
PRKACA	mRNA of PKA Catalytic Subunit C-alpha
MAOB	Monoamine oxidase [flavin-containing] B
PAQR6	Progesterone receptor
NCOA1	Nuclear receptor coactivator 2
PTGS1	Prostaglandin G/H synthase 1
VEGFA	Vascular endothelial growth factor A
HSP90	Heat shock protein 90
JUN	Transcription factor Jun
KCNC2	Potassium voltage-gated channel subfamily H member 2
BCL2L1	Bcl-2-like protein 1
DRD1	Dopamine D1 receptor
CHRM3	Muscarinic acetylcholine receptor M3
SCN5A	Sodium channel protein type 5 subunit alpha
GABRA2	Gamma-aminobutyric-acid receptor alpha-2 subunit
CHRM4	Muscarinic acetylcholine receptor M4
PDE3A	CGMP-inhibited 3′,5′-cyclic phosphodiesterase
HTR2A	5-hydroxytryptamine 2A receptor
GABRA5	Gamma-aminobutyric-acid receptor alpha-5 subunit
ADRA1A	Alpha-1A adrenergic receptor
GABRA3	Gamma-aminobutyric-acid receptor alpha-3 subunit
CHRM2	Muscarinic acetylcholine receptor M2
ADRA1B	Alpha-1B adrenergic receptor
ADRB2	Beta-2 adrenergic receptor
CHRNA2	Neuronal acetylcholine receptor subunit alpha-2
SERT	Sodium-dependent serotonin transporter
OPRM1	Mu-type opioid receptor
CHRNA7	Neuronal acetylcholine receptor protein, alpha-7 chain
CAMC	Cytochrome P450-cam
BCL2	Apoptosis regulator Bcl-2
BAX	Apoptosis regulator BAX
CASP9	Caspase-9
NAPA	Transcription factor AP-1
CASP3	Caspase-3
CASP8	Caspase-8
ATP1A1	Protein kinase C alpha type
TGFB1	Transforming growth factor beta-1
PON2	Serum paraoxonase/arylesterase 1
MAP 4	Microtubule-associated protein 2
NOS3	Nitric oxide synthase, endothelial
ADRA2A	Alpha-2A adrenergic receptor
SLC6A2	Sodium-dependent noradrenaline transporter
SLC6A3	Sodium-dependent dopamine transporter
LTA4H	Leukotriene A-4 hydrolase
MAOA	Amine oxidase [flavin-containing] A
CTRB1	Chymotrypsinogen B
ADRA2B	Alpha-2B adrenergic receptor
PLAU	Urokinase-type plasminogen activator
F2	Prothrombin
F9	Coagulation factor Xa
TOP2	DNA topoisomerase II
PTPN22	mRNA of Protein-tyrosine phosphatase, non-receptor type 1
RXRA	Retinoic acid receptor RXR-alpha
ESRRG	Estrogen receptor
PIM1	Proto-oncogene serine/threonine-protein kinase Pim-1
ACHE	Acetylcholinesterase

### Construction of PPI networks and identification of novel drug-disease core genes

**Fig. 3:**
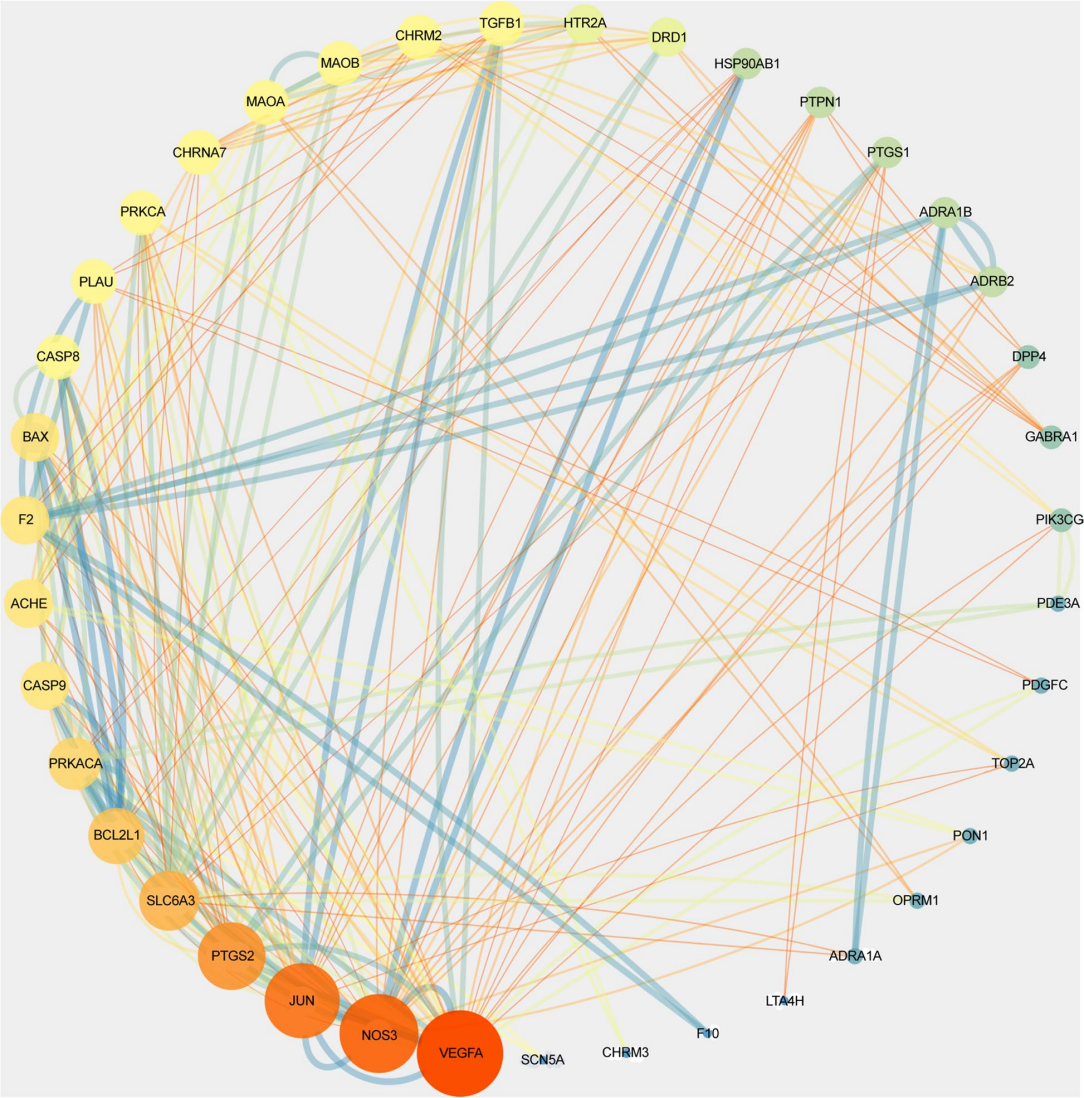
Protein-protein interaction network displayed by degree value

**Fig. 4:**
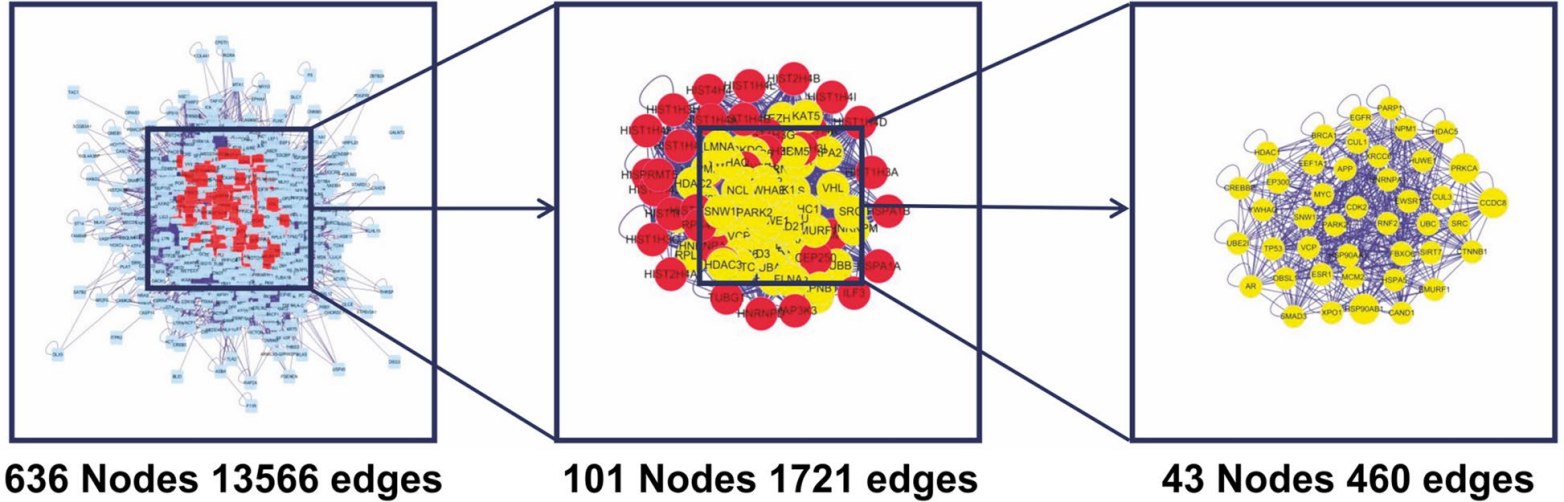
Screening process of drug-disease core genes

Each intersection gene was input into the STRING platform to obtain a PPI network of 67 nodes and 599 interaction lines. The average node degree was 17.9, and the local clustering coefficient was 0.62 (*P* < 0.01). The core genes were identified as VEGFA, JUN, and NOS3 (Figs. 3 and 4).

In the network interaction diagram, the nodes become larger with the increase in the number of edges.

### GO functional analysis and KEGG enrichment pathway analysis

**Fig. 5:**
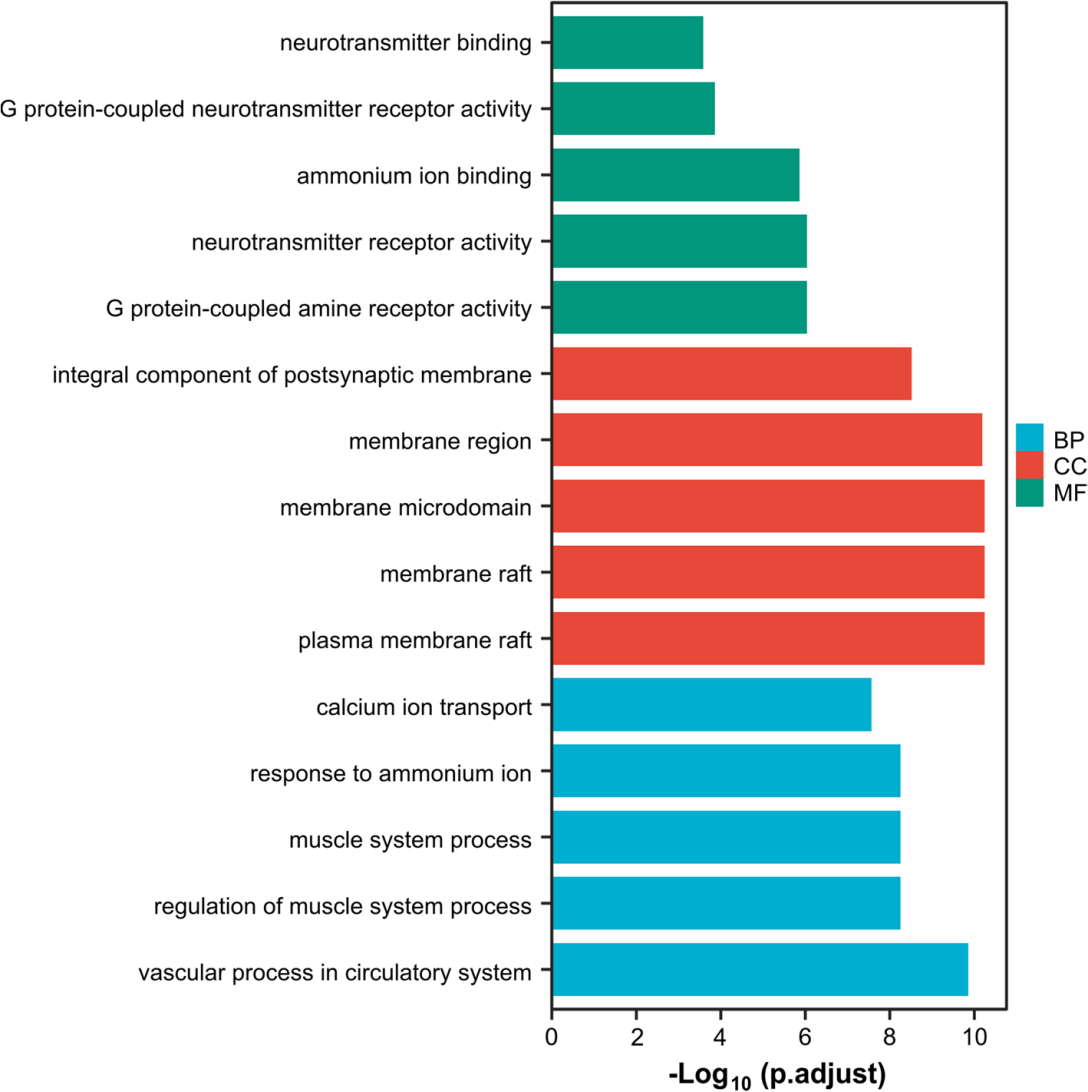
Gene Ontology enrichment analysis. BP, Biological process; CC, Cellular component; MF, Molecular function

Forty-three core genes were included in the GO enrichment analysis and the KEGG pathway (KEGG pathway comes from KEGG Database https://www.genome.jp/kegg/) enrichment analysis. A total of 761 biological processes were enriched, and 43 cell components and 50 molecular functions were obtained. The biological processes included the vascular processes in the circulatory system, regulation of muscle, muscle system processes, response to ammonium ion, and calcium ion transport. The cellular components included a plasma membrane raft, membrane raft, membrane microdomain, and membrane region. The molecular functions of an integral component of the postsynaptic membrane included G protein-coupled amine receptor activity, neurotransmitter receptor activity, ammonium ion binding, G protein-coupled neurotransmitter receptor activity, and neurotransmitter binding (Fig. 5).

**Fig. 6:**
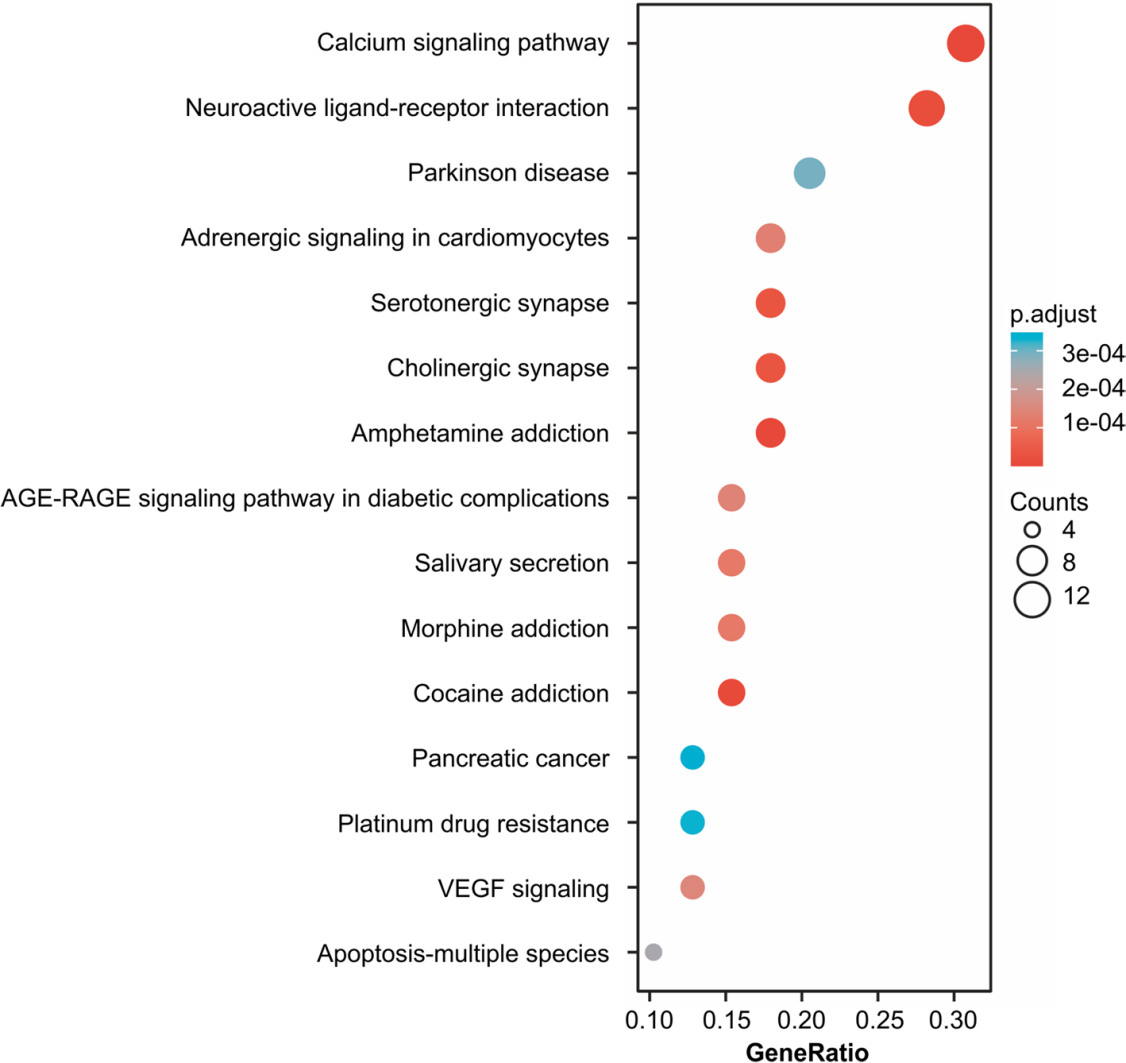
Kyoto Encyclopedia of Genes and Genomes pathway enrichment analysis. AGE-RAGE, Advanced glycation end products-recombinant advanced glycation end products; VEGF, Vascular endothelial growth factor

**Fig. 7:**
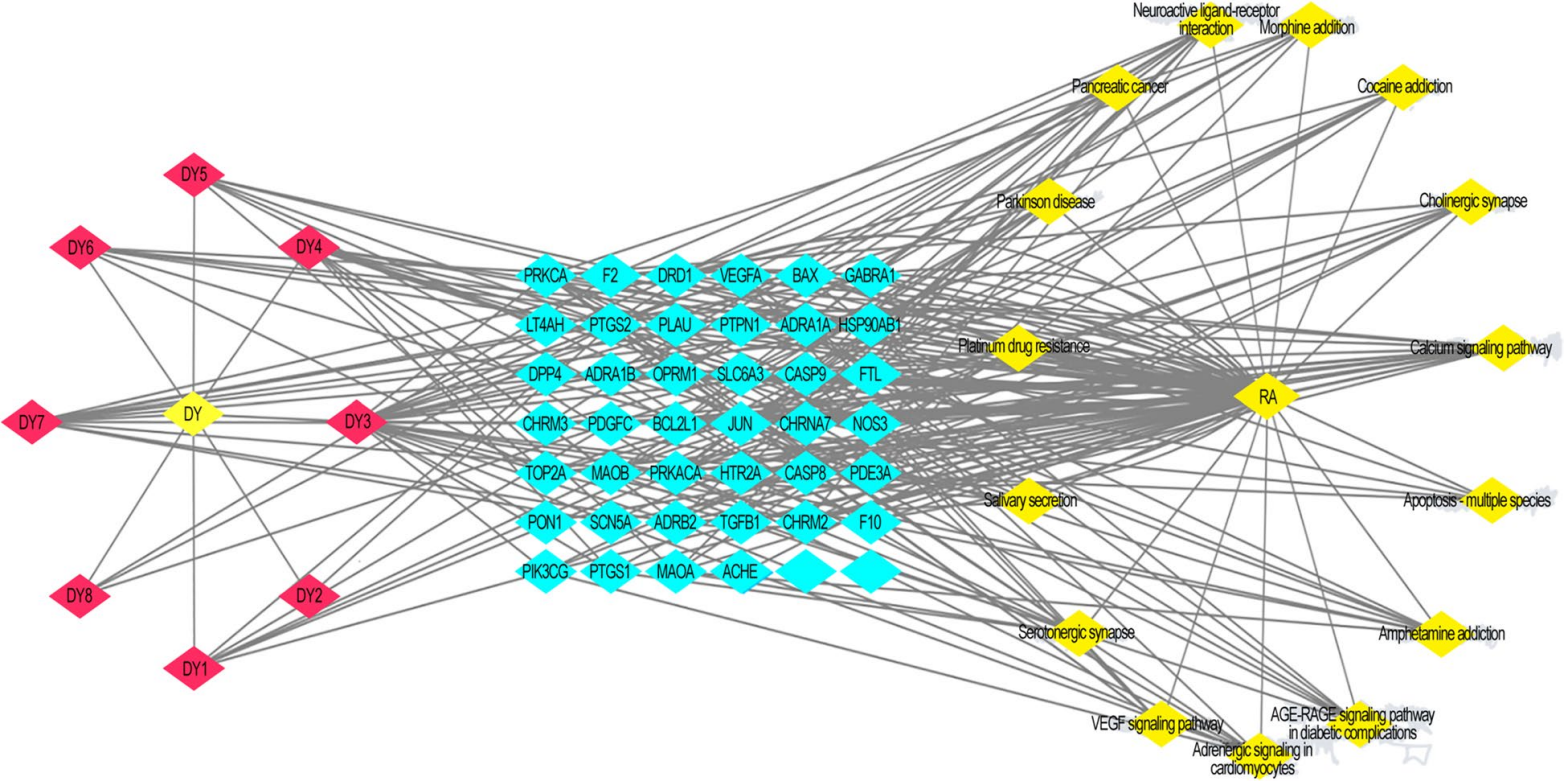
Drug-component-target-disease-pathway network

Fifteen core genes had a high correlation with *Angelicae Pubescentis Radix* in the KEGG pathway enrichment analysis. The core genes were mainly enriched in the pathways of calcium signaling, amphetamine addiction, cocaine addiction, neuroactive ligand-receptor interaction, cholinergic synapse, serotonergic synapse, morphine addiction, salivation, adrenergic signaling in cardiomyocytes, advanced glycation end products-recombinant advanced glycation end products (AGE-RAGE) signaling in diabetic complications, vascular endothelial growth factor signaling, apoptosis of multiple species, Parkinson’s disease, platinum drug resistance, and pancreatic cancer (Figs. 6 and 7).

Genes and proteins do not independently exhibit their own pharmacological and biological activities. Rather, they exert their effects through cellular and molecular pathways and shared networks. Accordingly, the use of interactive networks of disease/drug-related targets and signaling pathways are needed to clarify the molecular mechanism of *Angelicae Pubescentis Radix* in the treatment of RA. The network used in our study included 65 key nodes, across 8 active components of *Angelicae Pubescentis Radix*, with β-sitosterol identified as playing an important role. Among the 15 signaling pathways, the VEGF signaling pathway was considered a key pathway for the treatment of RA. Among the 40 potential targets of the VEGF signaling pathway, VEGFA, NOS3, JUN, PTGS2, SLC6A3, BCL2L1, PRKACA, CASP9, BAX, and F2 were considered to be high-degree targets for *Angelicae Pubescentis Radix* and, thus, important targets for the treatment of RA. These findings indicate that the mechanism of *Angelicae Pubescentis Radix* in the treatment of RA might be related to the regulation of cell proliferation by the key target proteins identified.

In the network interaction diagram, the yellow hexagon represents *Angelicae Pubescentis Radix*, and the red, blue, green, and yellow diamonds represent the active ingredient, target, KEGG pathway, and RA, respectively.

### Molecular docking technology analysis

**Fig. 8:**
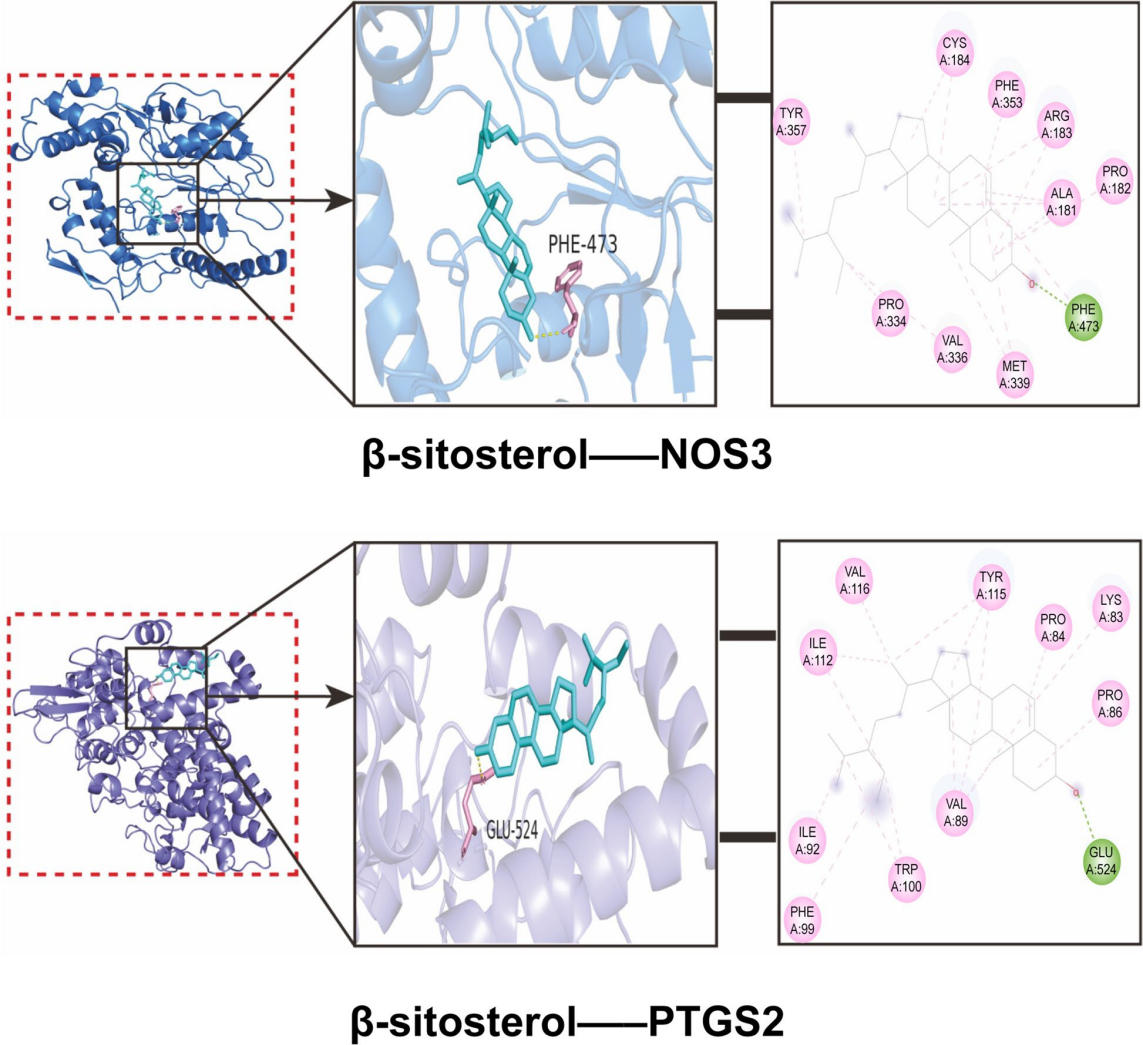
Interaction of β-sitosterol and receptor molecules. NOS3, Endothelial nitric oxide synthase 3; PTGS2, Prostaglandin endoperoxide synthase 2

The top 10 targets in the PPI network were included in the molecular docking analysis. The binding energy was > 4.52 kcal/mol. The molecular dockings of β-sitosterol and VEGFA (PDB ID: 1VPF), NOS3 (PDB ID: 1M9J), PTGS2 (PDB ID: 5F19), and SLC6A3 (PDB ID: 4XPF) were visualized. β-sitosterol was connected with the amino acid residue PHE-473 of the NOS3 gene via hydrogen bonds, while β-sitosterol and GLU-524 were connected via hydrogen bonds at the amino acid residue of PTGS2 (Table 3 and Fig. 8).

**Table 3:** Binding energy of β-sitosterol to targets in PPI network

Target	PDB ID	Affinity
VEGFA	1VPF	−6.1
NOS3	1M9J	−8.9
JUN	1A02	−5.4
PTGS2	5F19	−7.9
SLC6A3	4XPF	−7.8
BCL2L1	1YSN	−8
PRKACA	1CDK	−7.8
CASP9	2AR9	−6.6
BAX	1F16	−7.4
F2	7TPP	−7.6

Abbreviations: *PPI* Protein-protein interaction, *PDB* Protein Data Bank, *VEGFA* Vascular endothelial growth factor-A, *NOS3* Endothelial nitric oxide synthase, *JUN* Jun proto-oncogene, *PTGS2* Prostaglandin endoperoxide synthase-2, *SLC6A3* Solute carrier family 6 (neurotransmitter transporter), member 3; *BCL2L1* B-cell lymphoma-2-like protein 1, *PRKACA* Protein kinase A, *CASP9* Caspase-9, *BAX* BCL2-associated X, *F2* Coagulation factor II

### Signal path verification

**Fig. 9:**
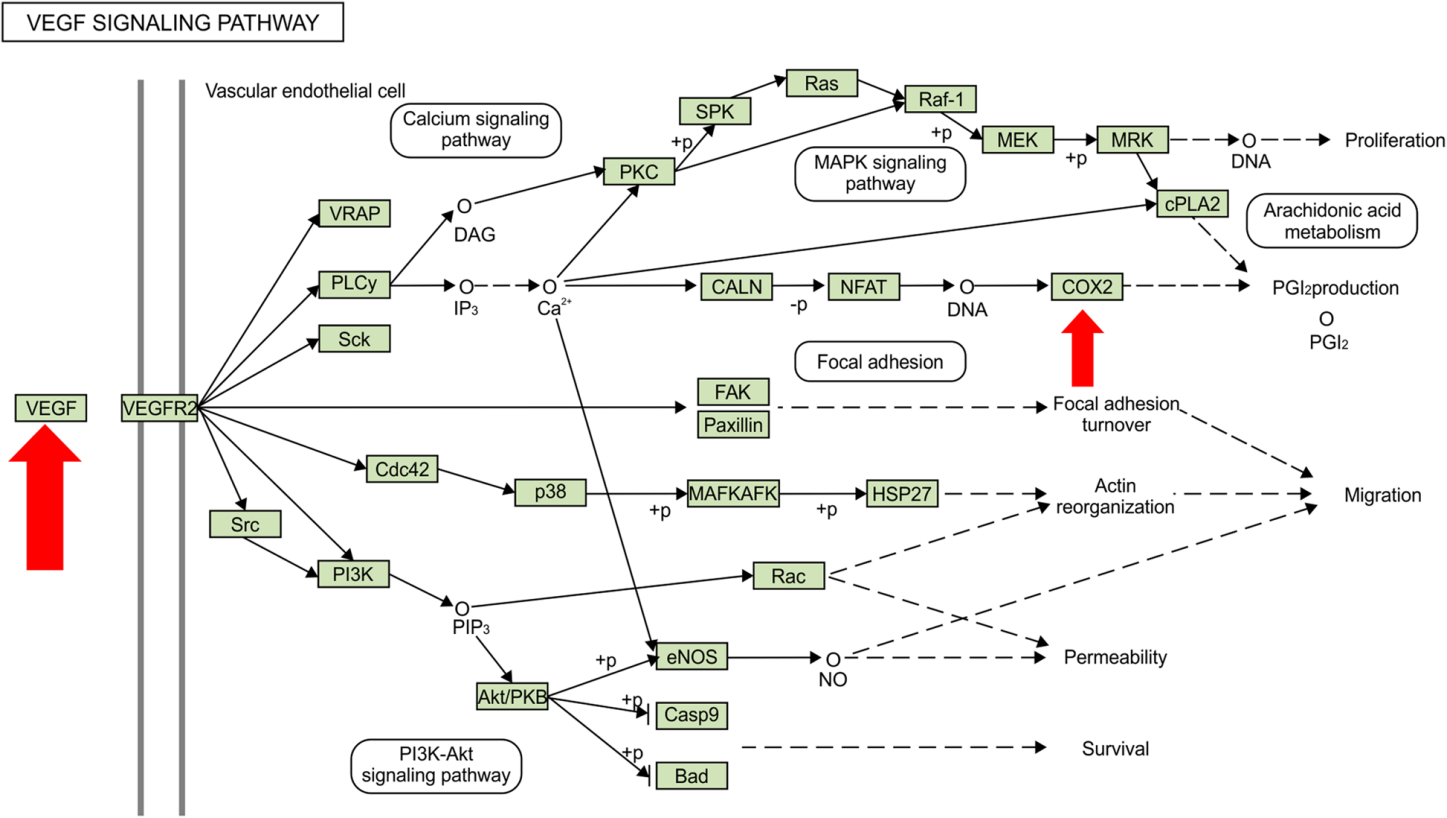
Cellular processes of VEGFA and PTGS2 in the VEGFA signaling pathway. VEGFA, Vascular endothelial growth factor A; PTGS2, Prostaglandin endoperoxide synthase 2; VEGFR2, Vascular endothelial growth factor receptor 2

PTGS2 was identified as an inflammatory factor in the VEGF pathway that promotes the production of PGI2. PTGS2 was identified as cyclooxygenase-2 (COX-2) in the KEGG database and network pharmacology, a key enzyme that promotes the synthesis of prostaglandins from arachidonic acid. Prostaglandins are involved in the inflammatory process (Fig. 9).

### Gene expression levels in human tissues

**Fig. 10:**
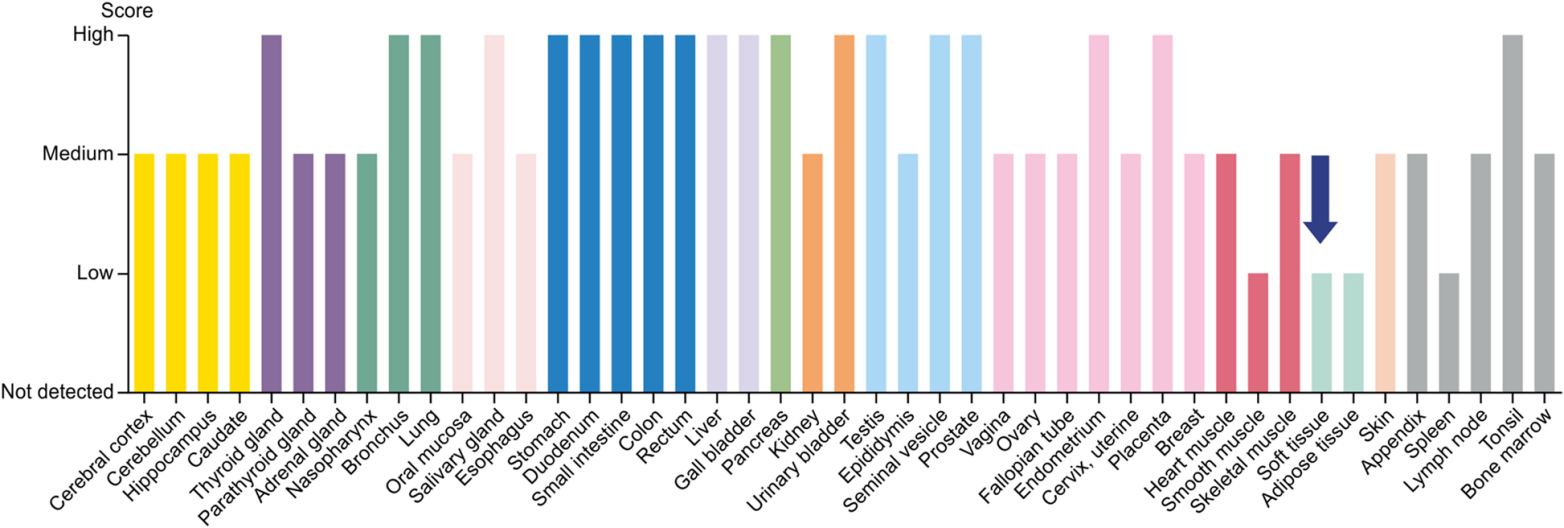
Expression of PTGS2 in various tissues. PTGS2, Prostaglandin endoperoxide synthase 2

The expression levels of NOS3 and PTGS2 in human tissues were obtained from the Human Protein Atlas database. PTGS2 was present and expressed in soft tissues, indicating that the expression of PTGS2 is related to the development of RA (Fig. 10).

### Cellular verification

#### β-Sitosterol inhibits the proliferation of MH7A cells

**Fig. 11:**
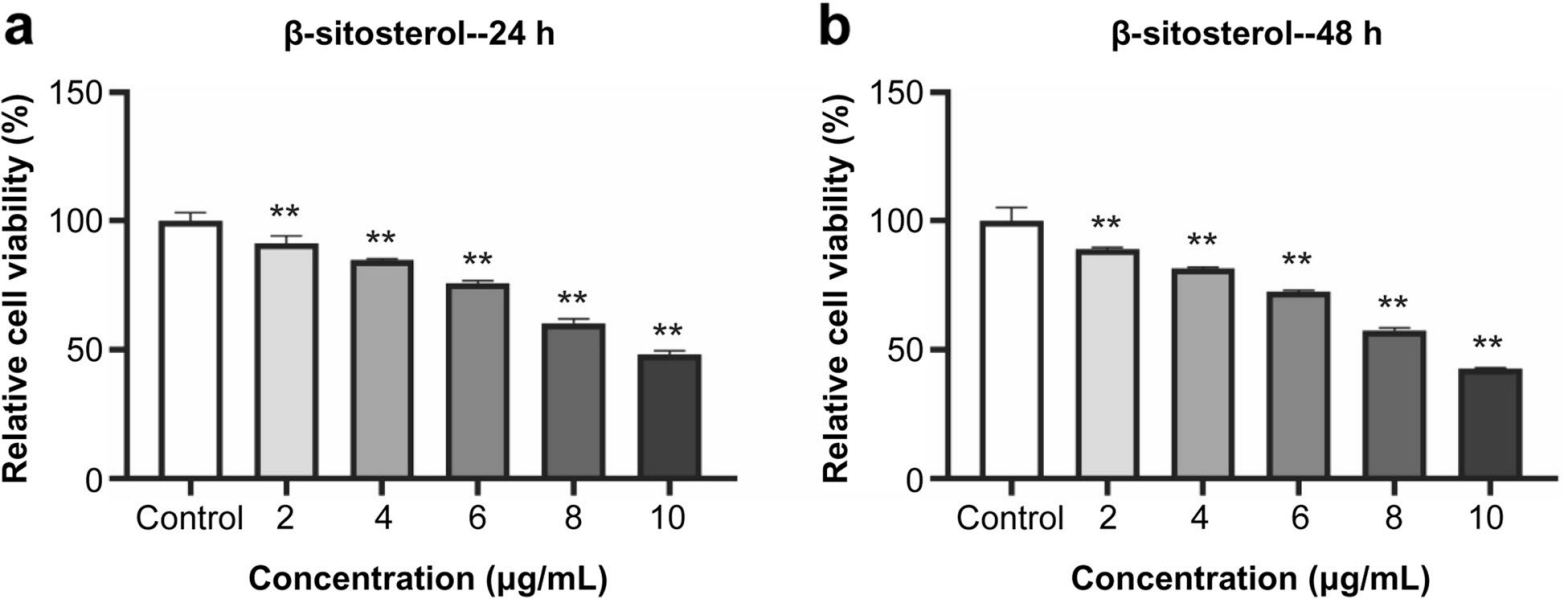
MTT assay to detect the toxicity of β-sitosterol on MH7A cells. **a** Relative cell viability after 24 hours. **b** Relative cell viability after 48 hours

The viability of MH7A cells was significantly lower than that of the control group after 24 h with 2 μg/mL β-sitosterol (*P* < 0.01) (Fig. 11(a)). MH7A cell viability decreased in a concentration-dependent manner. The cell viability also decreased in a time-dependent manner, as the cell viability was significantly lower after 48 h of treatment than after 24 h (*P* < 0.01) (Fig. 11(b)).

#### β-Sitosterol induces apoptosis in MH7A cells

**Fig. 12:**
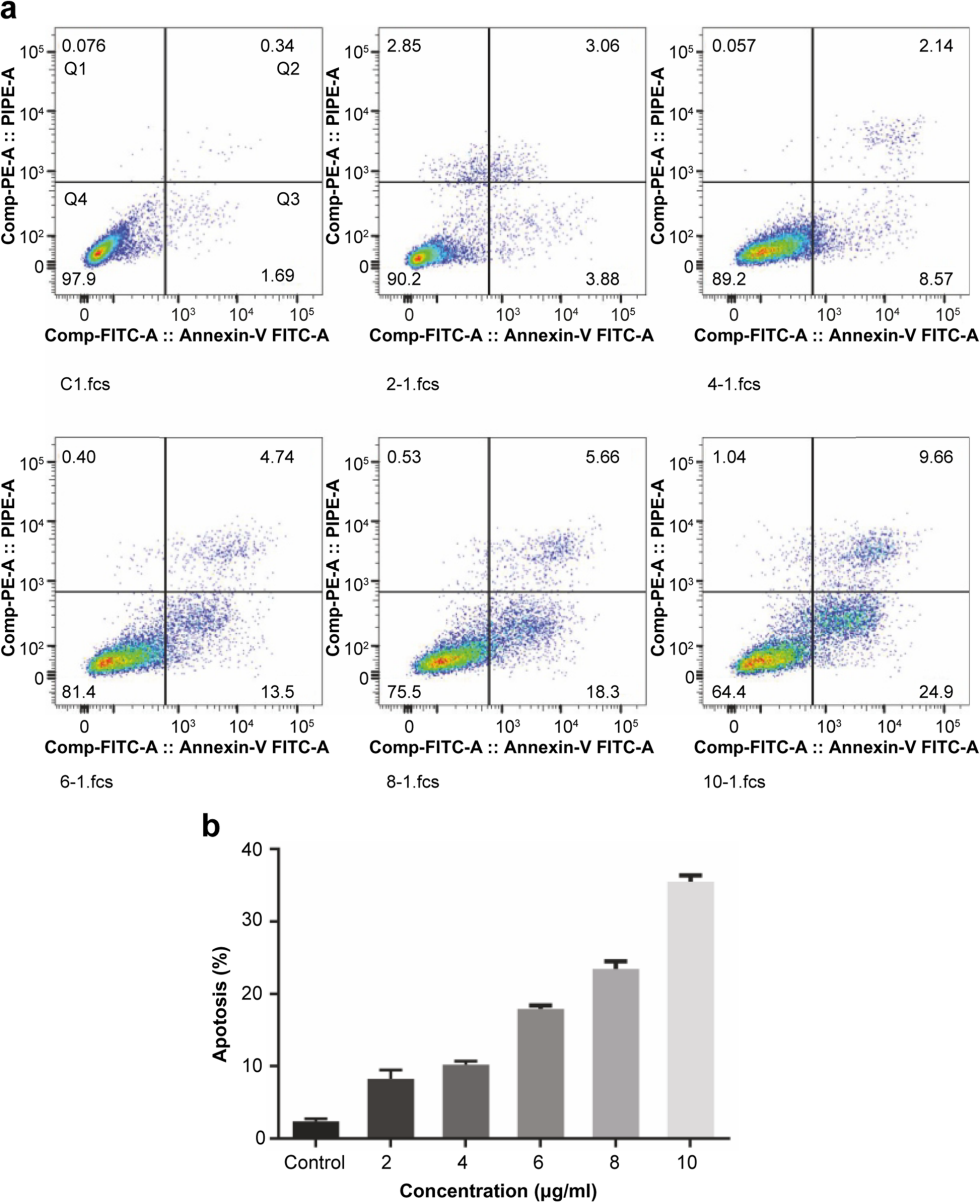
Apoptosis of MH7A cells. **a** Cell apoptosis after 24 hours. **b** Statistical analysis of apoptosis after 24 hours

To verify the effect of different drug concentrations on the apoptosis rate of MH7A cells, interestingly, we found that after treatment with β-sitosterol for 24 h, MH7A cells showed several signs of premature apoptosis, and the total apoptosis rate increased as the concentration of β-sitosterol increased (*P* < 0.05) (Fig. 12(a,b)), thus indicating that β-sitosterol promotes the premature apoptosis of cells and increases the total apoptosis rate to achieve the purpose of inhibiting the survival of RA cells and treating diseases.

#### β-Sitosterol induces S-phase retardation in MH7A cells

**Fig. 13:**
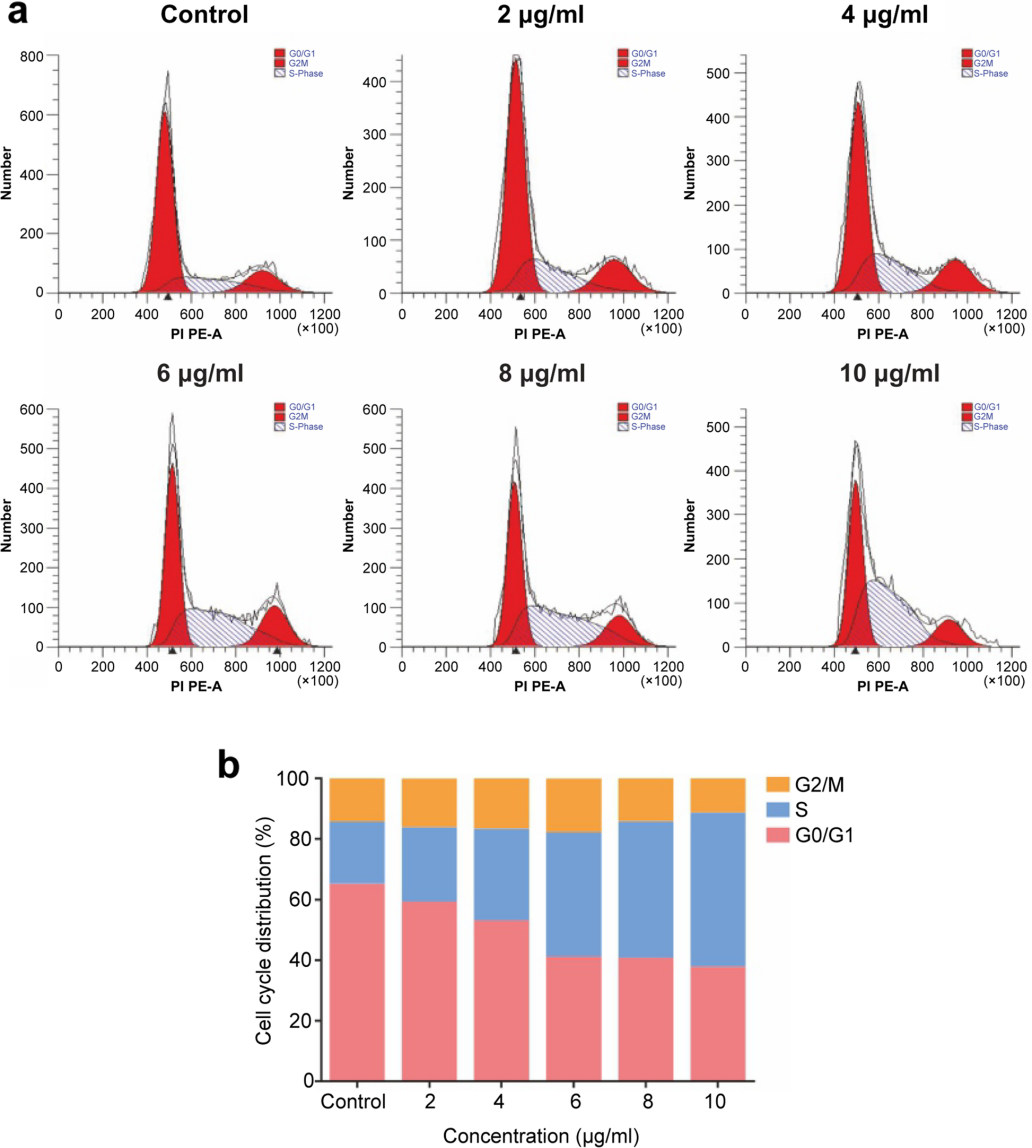
Cell cycle S-phase retardation diagram. **a** Cell cycle S-phase retardation change after 24 hours. **b** Statistical analysis of S-phase retardation after 24 hours

As the concentration of β-sitosterol increased, the distribution of MH7A cells in the S phase significantly increased (*P* < 0.05) (Fig. 13(b)), and the distribution in the G0/G1 phase decreased. β-sitosterol induced S-phase blockade in MH7A cells and promoted the transformation of G0/G1 phase cells to the S phase (Fig. 13(a)).

#### Effects of β-sitosterol on protein expression in MH7A cells

**Fig. 14:**
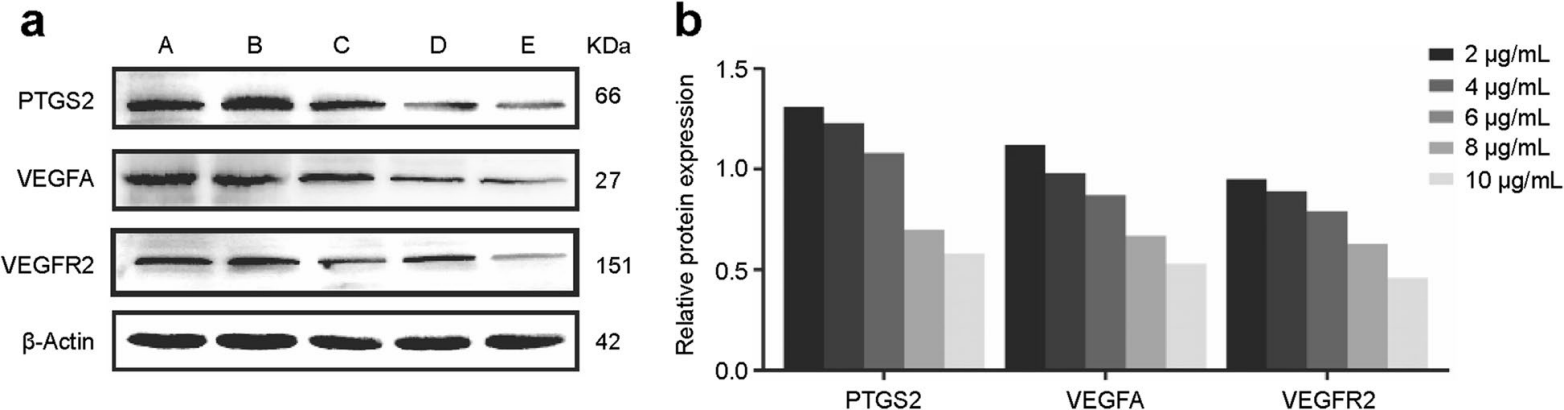
Protein expression levels in different treatment groups. **a** Western blots showing protein expression levels. **b** Statistical analysis of protein expression levels. A. 2 μg/mL β-sitosterol group, B. 4 μg/mL β-sitosterol group, C. 6 μg/mL β-sitosterol group, D. 8 μg/mL β-sitosterol group, E. 10 μg/mL β-sitosterol group

The expressions of PTGS2, VEGFA, and VEGFR2 proteins in MH7A cells decreased as the concentration of β-sitosterol increased (Fig. 14(a,b)).

## Discussion

RA is a chronic autoimmune disease that is related to several proteins and pathways in the process of disease development and is closely related to the oxidative stress response. During oxidative stress, the inflammatory factors produced in patients with RA further intensify the oxidative stress, which positively feeds back to increase inflammatory factors and continuously aggravates RA [13]. TCM has a variety of chemical components, and it has obvious curative effects on RA through multi-target and multi-channel comprehensive regulation abilities [14]. Network pharmacology is a discipline integrating biology and computer technology, which provides a new direction for studying the complex mechanism of TCM. We used this method to study the effect and mechanism of *Angelicae Pubescentis Radix* on RA and to conduct research experiments in vitro.

Based on the TCMSP database, compounds must have pharmacokinetic profiles to reach target organs for the delivery of biological activity. Compounds with OB ≥30% and DL ≥0.18 in *Angelicae Pubescentis Radix* have been considered to have significant pharmacokinetic activity in the current study [15]. In the present study, compounds with high biological activity were considered to be important in the mechanism of RA treatment, and β-sitosterol was the most representative compound, followed by imperatorin, isoimperatorin, and nodakenin. β-sitosterol has anti-inflammatory and immune regulatory effects because it reduces the levels of inflammatory factors in VEGFA and PTGS2. Isomers such as isoimperatorin and imperatorin have similar pharmacological, anti-inflammatory, and antibacterial effects. The efficacy of imperatorin is stronger than that of isoimperatorin, and imperatorin is a double inhibitor of epoxy enzyme and lipase. Previous studies have reported that imperatorin inhibits the production of prostaglandins in rat peritoneal macrophages and inhibits the production of epoxy enzyme-2 and prostaglandin synthetase. Imperatorin also helps cells resist various bacteria, such as gram-positive bacteria, *Escherichia coli*, *Staphylococcus aureus*, and *Pseudomonas aeruginosa* [16]. Imperatorin also has a significant antiviral effect. Imperatorin is used to treat arthritis as it reduces the levels of interleukin (IL)-1β, IL-6, and tumor cell necrosis factor-α inflammatory factors. Nodakenin reduces the levels of inflammatory factors such as IL-4, IL-5, and IL-13 and inhibits tracheal reactions [16]. All this literature, together with our experimental studies, supports the conclusion of network pharmacology prediction and proves that network pharmacology can be successfully applied to the study of the action mechanism of Chinese medicine.

From the perspective of integrated drug target prediction and pathway analysis, VEGFA, JUN, NOS3, and PTGS2 were identified as the core targets. Under hypoxic conditions, VEGFA increases by 30 times within a few minutes, resulting in the formation of new blood vessels. VEGFA promotes continuous proliferation of the synovial membrane and the generation of new blood vessels in pannus tissue, which are important causes of bone damage in patients with RA. JUN induces increased expression of Fre-1 and Fra-1 proteins in the periphery of macrophages by regulating the expression of pro-inflammatory factors and chemokines. Fra-1 induces an increase in IL-8. c-JUN is a component of transcriptional activator protein factor 1 (AP-1) and a marker of the proliferation and invasion of RA cells [17–19]. AP-1 regulates cell proliferation, differentiation, invasion, metastasis, and apoptosis. Studies have shown that c-JUN can be used as an important therapeutic target for acute inflammatory response and RA cells. NOS3 is an endothelial nitric oxide synthase (eNOS). To eliminate inflammation of RA, pro-inflammatory factors produce inducible nitric oxide synthase (iNOS) in large amounts. However, the synthesis of iNOS requires a large amount of arginine, decreasing the catalytic function of eNOS due to the lack of arginine. eNOS produces a low concentration of physiological nitric oxide, which is necessary for the functioning of several cellular mechanisms and pathways [20]. The PTGS2 gene encodes the production of COX-2, which is an important rate-limiting enzyme in the metabolism of arachidonic acid, prostaglandin, and thromboxane A2. Cyclooxygenase is divided into the following three subtypes: COX-1, COX-2, and COX-3. COX-2 is an inducible enzyme that is expressed at very low levels in normal tissue cells and very high levels in inflammatory tissues under the stimulation of inflammatory cytokines [21]. The high expression of COX-2 leads to a significant production of prostaglandins (PEG2). PEG2 is an important inflammatory mediator that sensitizes pain nerves, dilates blood vessels, and aggravates inflammation. The causes of RA have been reported as the infiltration and invasion of inflammatory cells and the pathological neovascularization of the synovial membrane. COX-2 and PEG2 are abundant in inflammatory tissues, and their combined actions enhance the inflammatory response and the damage to bone and cartilage. Therefore, PTGS2 is an important target for the treatment of RA. The relationship between β-sitosterol and the target genes NOS3 and PTGS2 was elucidated from the molecular structure using molecular docking technology in this study. PTGS2 is highly expressed in soft tissues and can be used as a marker gene for RA.

As predicted by network pharmacology, *Angelicae Pubescentis Radix* may exert therapeutic effects on RA mainly by regulating cell proliferation and cell survival of RA through the VEGF signaling pathway. The VEGF signaling pathway works with several other pathways to regulate the angiogenesis process and is the most important pathway to promote neoangiogenesis and generate pannus. Therefore, the VEGF signaling pathway is a landmark pathway to clarify the pathogenesis of RA and monitor the development of RA.

To further verify this hypothesis, we studied the therapeutic effect of β-sitosterol on RA cells in vitro. β-sitosterol significantly inhibited the activity and proliferation of RA cells, reduced the distribution of the G0/G1 phase of cells, and promoted the transformation of cells to the S phase. The results of Western bolt showed that β-sitosterol inhibited the proliferation and invasion of RA, significantly reduced the expression of PTGS2, VEGFA, and VEGFR2 proteins in a dose-dependent manner, and inhibited the VEGF signaling pathway. The VEGF signaling pathway has been confirmed to mediate endothelial cell proliferation, migration, and anti-apoptosis and improve vascular permeability. The high expression of VEGF in joint fluid and in the serum of patients with RA indicates that it is related to the progression of RA [22]. Taken together, it has been proved in the present study that β-sitosterol can be used to downregulate PTGS2, VEGFA, and VEGFR2 via the VEGF signaling pathway to induce the apoptosis of RA cells, and further detailed pharmacological mechanisms will be explored in future studies.

This study was not without limitations. As the public database is updated in real-time, the mechanism of β-sitosterol in the treatment of RA is partially unclear. In this study, human RA cells were used. Therefore, multi-dimensional and multi-faceted studies are required to fully explore the mechanism of β-sitosterol in the treatment of RA. Although β-sitosterol is the main active component of *Angelicae Pubescentis Radix,* it is not the only active component. Therefore, further verification of other ingredients is required.

## Conclusions

This study determined the complex relationship between the components, targets, and pathways of *Angelicae Pubescentis Radix* used to treat RA. The roles of the core targets and signaling pathways for regulating immunity, providing anti-inflammatory effects, and inhibiting inflammatory factors have been clarified. The identification of the key targets of *Angelicae Pubescentis Radix* during the treatment of RA allows for further elucidation of its pharmacological effects, which will improve the treatment methods for RA.

## Supplementary Information

**Additional file 1:** **Figure S1.** Original picture of protein expression level.

## Data Availability

The datasets used and/or analyzed during the current study are available from the corresponding author upon reasonable request.

## Abbreviations

ADME: absorption, distribution, metabolism, and excretion
AGE-RAGE: Advanced glycation end products-recombinant advanced glycation end products
AP-1: Activator protein 1
BAX: BCL2-associated X
BCA: Bicinchoninic acid
BCL2L1: B-cell lymphoma-2-like protein 1
CASP3: Caspase-3
CASP9: Caspase-9
COX-1: COX-2, COX-3; Cyclooxygenase-1, Cyclooxygenase-2, Cyclooxygenase-3
DL: drug-like
DMSO: Dimethylsulfoxide
ECL: Electrochemiluminescence
eNOS: Endothelial nitric oxide synthase
F2: Coagulation factor II
Fra-1: Fos-related antigen-1
Fre-1: Ferric-chelate reductase 1
GeneCard: Human gene database
GO: Gene Ontology
IL-1β, IL-4, IL-5, IL-6, IL-8, IL-13: Interleukin-1β, Interleukin-4, Interleukin-5, Interleukin-6, Interleukin-8, Interleukin-13
iNOS: Inducible nitric oxide synthase
Jun: Jun proto-oncogene
KEGG: Kyoto Encyclopedia of Genes and Genomes
MTT: 3-(4,5-Dimethylthiazol-2-yl)-2,5-diphenyl-2H-tetrazolium bromide
NOS3: Nitric oxide synthase 3
OB: Oral bioavailability
OD: Optical density
OMIM: Online Mendelian Inheritance in Man
PBS: Phosphate buffer saline
PDB: Protein Data Bank
PEG2: Prostaglandin E2
PPI: Protein-protein interaction
PRKACA: Protein kinase A
PTGS2: Prostaglandin endoperoxide synthase 2
RA: Rheumatoid arthritis
SD: Standard deviation
SLC6A3: Solute carrier family 6 (neurotransmitter transporter), member 3
TBST: TBS-Tween®
TCM: Traditional Chinese medicine
TCMSP: Traditional Chinese medicine systems pharmacology
Uniprot: The global protein resource database
VEGFA: Vascular endothelial growth factor-A
VEGFR2: Vascular endothelial growth factor receptor 2
