# Supplementary figures and images for: Computation and molecular pharmacology to trace the anti-rheumatoid activity of Angelicae Pubescentis Radix
Source: BMC Complement Med Ther. 2022 Nov 26;22:312. doi: 10.1186/s12906-022-03769-w (PMC9701395; doi:10.1186/s12906-022-03769-w)

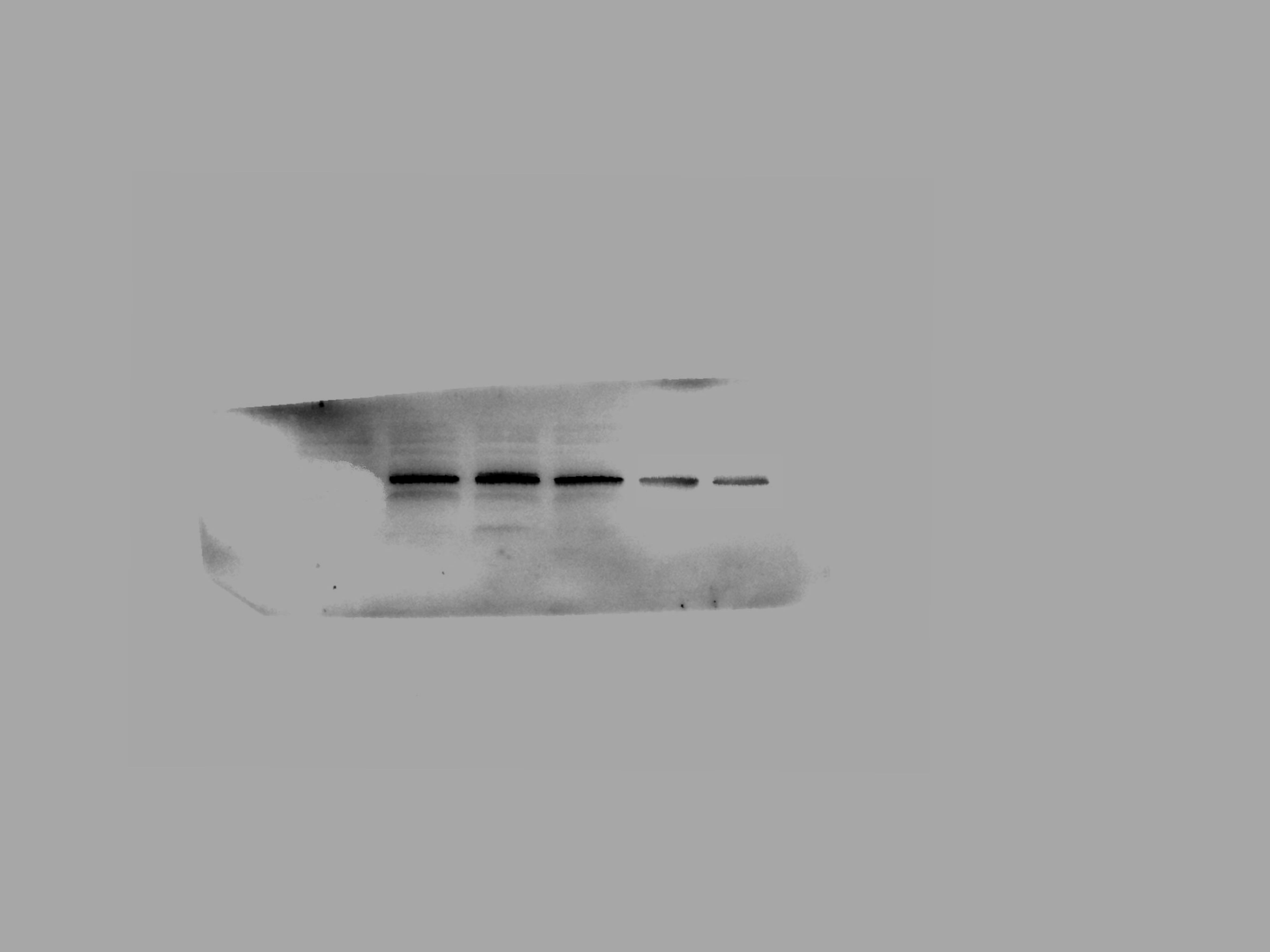

Supplement: Supplementary file 1 — Additional file 1: Figure S1. Original picture of protein expression level. [file 12906_2022_3769_MOESM1_ESM.zip › S1-1 Strip picture.tif]

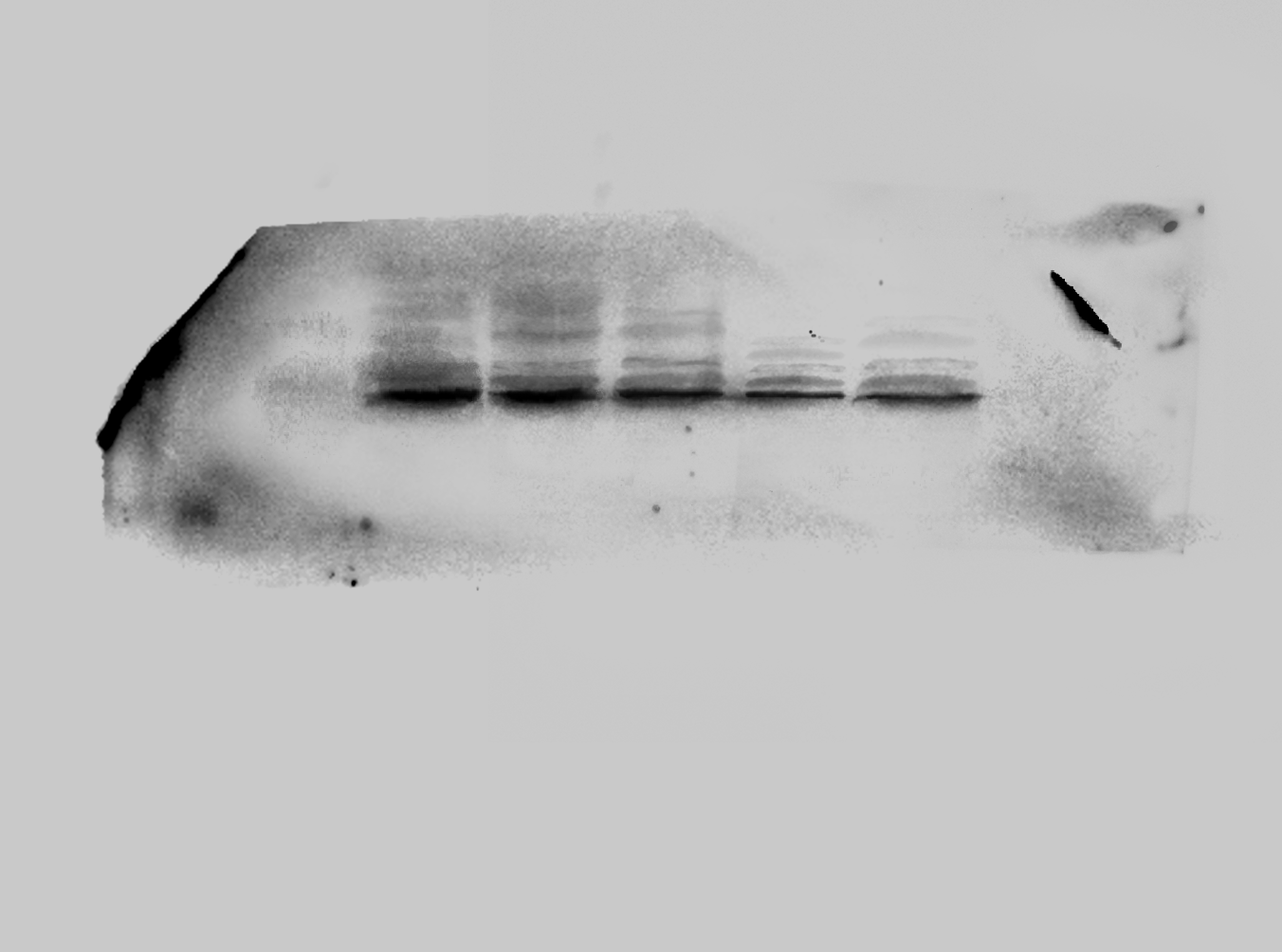

Supplement: Supplementary file 1 — Additional file 1: Figure S1. Original picture of protein expression level. [file 12906_2022_3769_MOESM1_ESM.zip › S1-2 VEGFA Strip picture.tif]

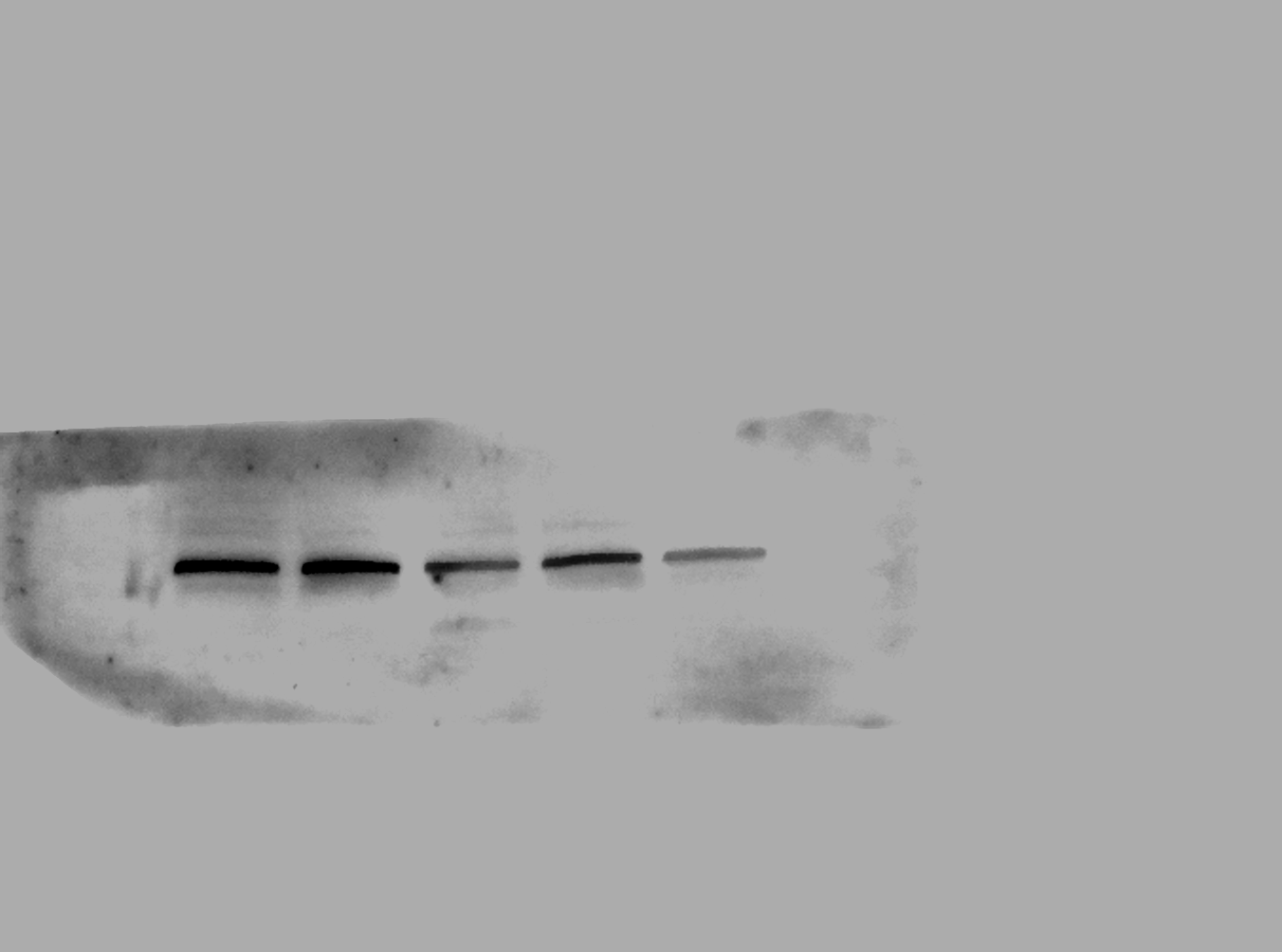

Supplement: Supplementary file 1 — Additional file 1: Figure S1. Original picture of protein expression level. [file 12906_2022_3769_MOESM1_ESM.zip › S1-3 VEGFR2 Strip picture.tif]

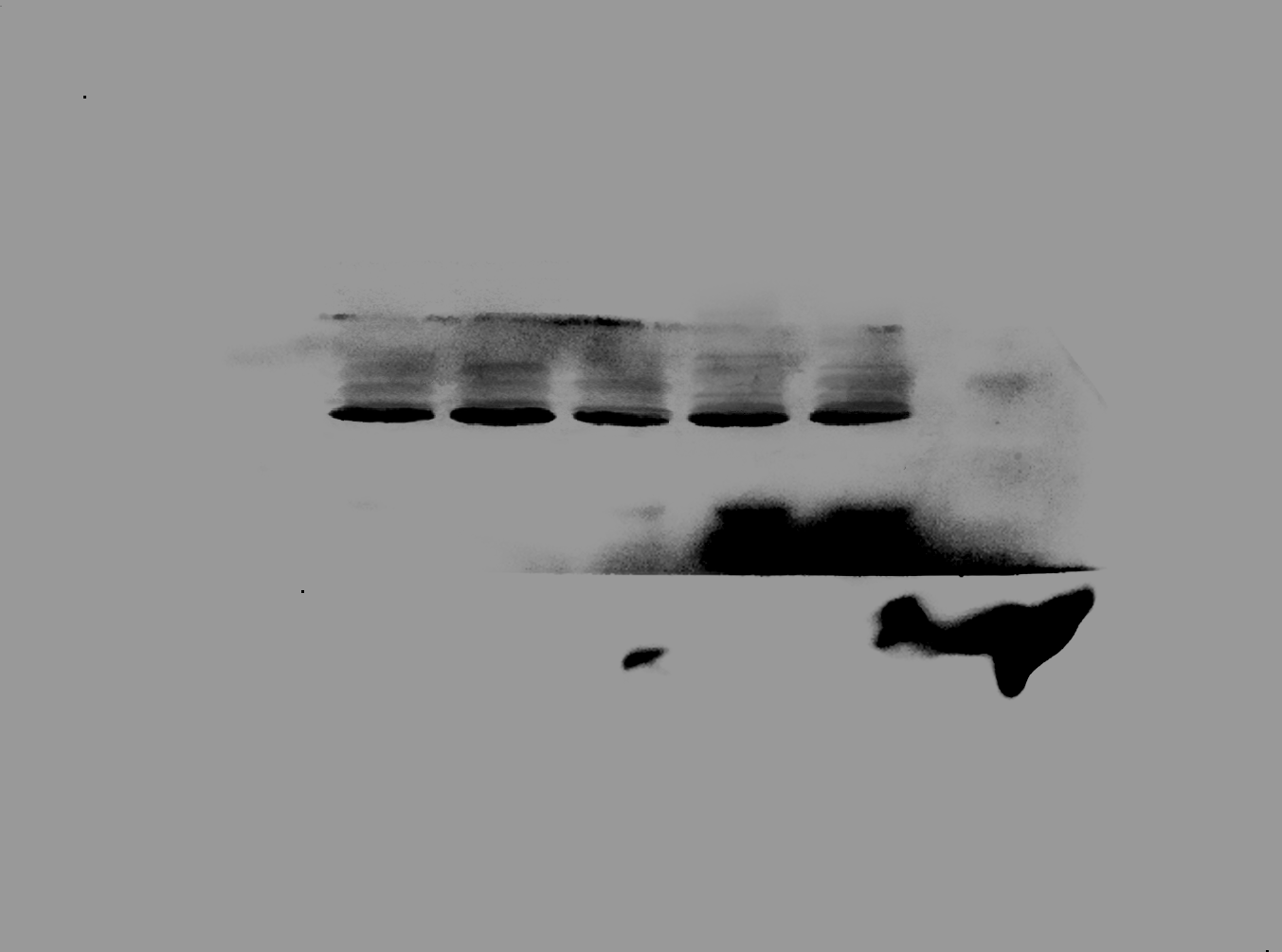

Supplement: Supplementary file 1 — Additional file 1: Figure S1. Original picture of protein expression level. [file 12906_2022_3769_MOESM1_ESM.zip › S1-4 Strip picture.tif]
